# Efficacy and Safety of Stunnox™ (Botulinum Toxin Type A) for the Treatment of Glabellar Frown Lines and Forehead Lines: A Prospective Observational Study in an Indian Cohort

**DOI:** 10.7759/cureus.118681

**Published:** 2026-10-27

**Authors:** Sandeep Arora, Gulhima Arora, Rajesh S Lalchandani

**Affiliations:** 1 Dermatology, Mehektagul Dermaclinic, New Delhi, IND; 2 Dermatology, Apollo Hospitals, New Delhi, IND; 3 Medical Affairs, Gufic BioSciences, Mumbai, IND

**Keywords:** botulinum toxin type a, cosmetic dermatology, facial wrinkle scale, forehead lines, glabellar frown lines, indian skin, stunnox

## Abstract

Background

Botulinum toxin is the most widely performed nonsurgical aesthetic procedure globally, but efficacy data in Indian skin phototypes remain limited.

Objective

This study aims to evaluate the efficacy (primary endpoint: complete resolution at week 1) and safety of Stunnox™ (botulinum toxin type A (BoNT-A); Gufic Biosciences, India) in Indian adults with Fitzpatrick III-V skin and grade 1-2 glabellar frown lines (GFL) and forehead lines (FL) over 20 weeks.

Methods

In this prospective, single-center, single-arm, open-label, investigator-initiated study, 77 Fitzpatrick III-V adults received Stunnox™ (BoNT-A; Gufic Biosciences, India) for GFL (individualized at the injector’s discretion, 9-17 U; mean 12.4 U) and FL (fixed 20 U). Fifty patients completed the full 20-week follow-up (mean age: 32.7 ± 5.7 years) and were scored on the Facial Wrinkle Scale (FWS) (0-3, dynamic expression) at baseline and post-treatment weeks 1, 4, 8, 12, 16, and 20. Analysis was restricted to the completers (50/77; 64.9%).

Results

Complete resolution was observed at the first assessment (week 1) for both regions (GFL: 1.64 to 0; FL: 1.18 to 0; p < 0.0001), sustained through week 12 (GFL) and 16 (FL). Partial recurrence appeared by week 20 (GFL 1.02; FL 0.96), still significantly below baseline (p < 0.001); one patient (2%) had early GFL recurrence. No serious adverse events occurred; five patients had transient brow heaviness for four weeks without ptosis. By week 20, 38% (GFL) and 78% (FL) of patients had returned to their baseline grade. Exploratory severity-based glabellar dosing (Grade 1: 10.7 U; Grade 2: 13.3 U) was used without a fixed-dose comparator.

Conclusions

Stunnox™ showed promising early efficacy and short-term tolerability for GFL and FL in Indian adults, with partial recurrence by week 20.

## Introduction

Facial wrinkles of the upper face are among the most common cosmetic concerns encountered in dermatological practice. Botulinum toxin type A (BoNT-A) injection is established as the gold standard nonsurgical treatment for upper-face dynamic wrinkles, the glabellar frown lines (GFL) and the forehead lines (FL) [1,2]. It represents the most frequently performed cosmetic procedure worldwide [3].

While the efficacy and safety of BoNT-A formulations have been extensively characterized in predominantly Caucasian cohorts, data in South Asian populations with Fitzpatrick phototypes III-V remain limited. Skin biophysical characteristics, muscular anatomy, and expression patterns vary by ethnicity [4], highlighting the need for region-specific outcome data. Further, the majority of published trials use fixed, protocol-mandated dosing regimens, which may not reflect individualized clinical practice [5,6].

Interest has also grown in the early (preventive) use of BoNT-A in younger adults with mild dynamic wrinkles; however, the evidence that this prevents the formation of static rhytids remains limited and heterogeneous [7]. Repeated muscular contraction over the years causes eventual transition from dynamic to static furrows that are refractory to neuromodulator therapy alone. By interrupting this cycle early, preemptive BoNT-A treatment can attenuate or delay the formation of static wrinkles [7,8]. The young age profile of our cohort (18-40 years) makes this study directly relevant to the preemptive treatment paradigm. However, the present study did not assess prevention; the age range of our cohort (18-40 years) is noted only as context.

Stunnox™ (Gufic Biosciences Ltd., India) is a locally manufactured BoNT-A formulation (Strain: Prime Bio USA MCL 44) that has gained regulatory approval in India [9]. However, the prospective clinical data characterizing its onset, magnitude, and duration of effect in a real-world Indian dermatology setting are limited.

This study aimed to evaluate the efficacy and safety of Stunnox™ over 20 weeks, after a single treatment, in Indian adults (Fitzpatrick phototypes III-V) with Grade 1-2 GFL and FL. The primary objective was to determine the proportion of patients achieving complete resolution (Grade 0 on the Facial Wrinkle Scale, FWS) at week 1. The secondary objectives were to describe the mean FWS grade at each time point, the duration of complete resolution, the time to recurrence, and the incidence of adverse events. We describe the outcomes of an individualized, severity-based glabellar dosing approach, for which there was no comparison group.

## Materials and methods

Study design and setting

This was a prospective, single-center, open-label, single-arm, noncomparative, investigator-initiated observational study (sponsor-funded) conducted as per the Declaration of Helsinki and the Good Clinical Practice guidelines, registered with the Clinical Trial Registry-India (CTRI/2024/12/077888). The study was conducted between January and August 2025. Ethical approval was obtained from the Risaa IVF Ethics Committee (approval number: RISAA/IEC/2024/11/30, dated 29 November 2024), and written informed consent was obtained from all participants prior to enrollment.

Participants

Adults aged 18 to 40 years presenting to a tertiary dermatology clinic seeking treatment for GFL and FL were screened. Of 179 individuals screened, 77 were enrolled; 50 completed the full 20-week follow-up and constitute the per-protocol population (Figure 1). The study was designed to obtain 50 participants who completed the 20-week follow-up. Because of the long follow-up period, an attrition of 20% was anticipated. Sixty-three participants were planned for enrollment. Attrition was higher than anticipated: 27 of the 77 participants enrolled and treated (35.1%) did not complete follow-up. Enrolment was therefore continued until 50 participants had completed the 20-week follow-up, as shown in the Consolidated Standards of Reporting Trials (CONSORT) diagram (Figure 1). The final completed cohort comprised 26 males and 24 females. The study was not powered for between-sex comparisons.

**Figure 1:**
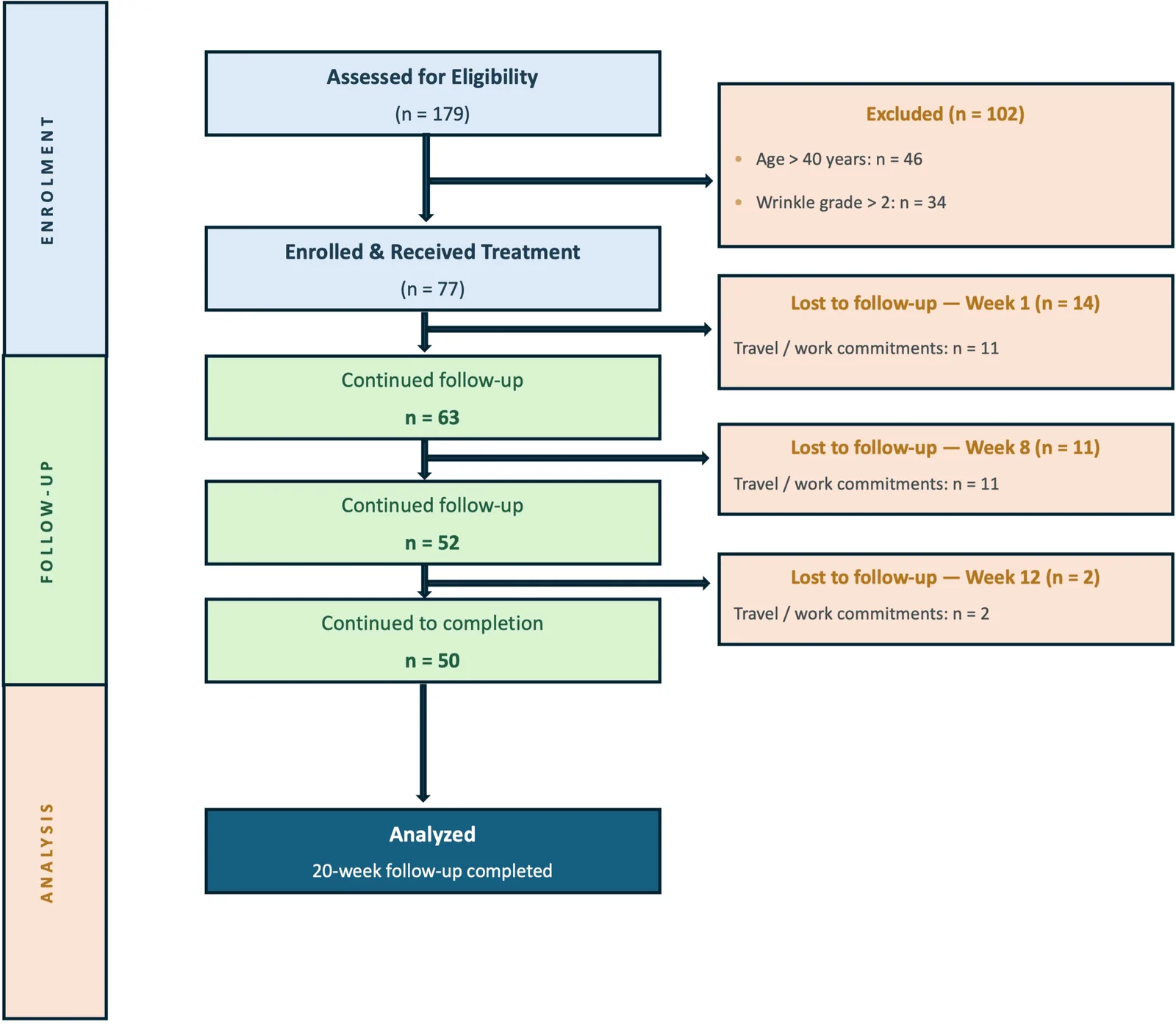
CONSORT flow diagram CONSORT: Consolidated Standards of Reporting Trials

Inclusion criteria included age 18-40 years, Fitzpatrick skin types III-V, Indian ethnicity, GFL and FL Grade 1 or 2 on the FWS [10,11], and a body mass index of <35 kg/m². Exclusion criteria were known hypersensitivity to BoNT-A, neuromuscular disorders, concomitant use of medications that interfere with neuromuscular transmission within four weeks prior to screening, prior BoNT-A injection to the forehead or glabella within four months, and pregnancy or lactation.

Treatment protocol

Stunnox™ was reconstituted with sterile preservative-free 0.9% sodium chloride solution in 50-unit vials, yielding a concentration of 4 U per 0.1 mL (1.25 mL of diluent for the 50U vial). FL received a fixed 20 U dose in five injection points (4 U/0.1 mL per site) into the occipitofrontalis muscle using the modified "M" injection pattern. GFL dosing was individualized based on baseline wrinkle severity across five injection points, two in each Corrugator Supercilii and one in the Procerus muscle, with the needle bevel facing upward. All injections were performed intramuscularly by a trained dermatologist using a 31-G needle. Injection points followed the corrugator supercilii, procerus, and frontalis targets described in published anatomical injection guidelines [1,2]; the forehead points were placed in the M pattern, 1.5-2 cm above the eyebrow to reduce the risk of brow ptosis [2]. The total glabellar dose (9-17 U) was left to the discretion of the treating dermatologist, guided by baseline severity and muscle bulk; the protocol set a maximum glabellar dose of 20 U [10] but did not prescribe a fixed dosing algorithm or per-site dose.

Efficacy assessments

Eligibility screening, including confirmation of GFL/FL grade on the FWS, was performed prior to enrollment to identify eligible patients; formal efficacy assessment records were maintained from the day of Stunnox™ administration (day 0/baseline) onward. Wrinkle severity was independently assessed at seven time points: baseline and post-treatment weeks 1, 4, 8, 12, 16, and 20, from standardized photographs (iPhone 15 Pro Max or DSLR) in frontal and bilateral 45-degree oblique views at rest and dynamic (animated) expression. FWS grading at each time point was performed on the dynamic (animated) expression images; at-rest images were retained for documentation and safety review only and were not separately scored. Patients were asked to frown maximally (glabella) and to raise the eyebrows maximally (forehead) for the dynamic images. The FWS is an ordinal four-point scale (0 = none, 1 = mild, 2 = moderate, 3 = severe) originally described for glabellar and forehead rhytids [10,11]. Grade 0 was defined as complete resolution, and improvement as a reduction of at least one grade from baseline, the responder definition commonly used in FWS-based studies [12,13]. Grading was performed by a second trained dermatologist, who was blinded to the injected dose and to the follow-up time point of each image set. The follow-up schedule (weeks 1, 4, 8, 12, 16, and 20) was fixed by the protocol, which also included a screening visit one week before treatment. 

The primary efficacy endpoint was the proportion of patients achieving Grade 0 (complete resolution) on the FWS at week 1 for GFL and FL. Secondary endpoints included the mean FWS grade at each time point, the duration of complete resolution, the time to onset of recurrence (the first visit at which the FWS grade exceeded 0), and the incidence of adverse events. The proportion of patients with at least a one-grade improvement from baseline at each time point (responder rate) is reported as an additional descriptive analysis.

Safety assessments

Adverse Events

Injection-site reactions (erythema, edema, bruising, pain), systemic events, and unintended effects (ptosis, brow asymmetry, dyskinesia) were recorded at all visits. Those who withdrew were contacted to record any side effects.

Statistical analysis

Descriptive statistics are expressed as mean ± standard deviation (SD) or as count and percentage. Changes in FWS score from baseline were assessed using the Wilcoxon signed-rank test for ordinal repeated-measures data. Gender-stratified FWS scores at week 20 were compared using the Mann-Whitney U test. Statistical significance was set at p < 0.05. All analyses were performed using Python (SciPy library, v1.11). Post-baseline comparisons were made separately for GFL and FL (12 Wilcoxon comparisons against baseline) without adjustment for multiplicity, so p-values are nominal; the conclusions are unchanged if a Bonferroni-adjusted threshold (0.05/12 = 0.004) is applied. Between-sex and other baseline comparisons (Table 1) were exploratory. Ninety-five percent confidence intervals were calculated using the t-distribution for means and the exact (Clopper-Pearson) method for proportions. Efficacy analyses were restricted to the 50 participants who completed follow-up (completers-only); data from the 27 noncompleters were not included, and no imputation was performed. 

**Table 1:** Baseline patient demographics and wrinkle characteristics (n = 50) FWS: Facial Wrinkle Scale; GFL: glabellar frown lines; FL: forehead lines; SD: standard deviation

Characteristic	Overall (N = 50)	Male (n = 26)	Female (n = 24)
Age, mean ± SD (years)	32.7 ± 5.7	34.2 ± 4.5	31.0 ± 6.4
Age range (years)	21-40	27-40	21-40
Fitzpatrick skin type, n (%)	III-V	-	-
Glabella: Grade 1, n (%)	18 (36%)	5 (19%)	13 (54%)
Glabella: Grade 2, n (%)	32 (64%)	21 (81%)	11 (46%)
Forehead: Grade 1, n (%)	41 (82%)	23 (88%)	18 (75%)
Forehead: Grade 2, n (%)	9 (18%)	3 (12%)	6 (25%)
Mean baseline GFL FWS	1.64 ± 0.48	1.81	1.46
Mean baseline FL FWS	1.18 ± 0.39	1.12	1.25
Glabella dose, mean ± SD (U)	12.4 ± 2.0	13 ± 2.2	11.8 ± 1.7
Forehead dose (all patients)	20 U (fixed)	20 U	20 U

Dosage administered

All participants received a fixed dose of 20 U for FL, and a dose range of 9 to 17 U (mean 12.4 ± 2.0 U) for GFL. Grade 1 patients received a mean dose of 10.7 ± 0.9 U, while Grade 2 patients received 13.3 ± 1.9 U. The most commonly administered dose was 12 U (n = 15; 30%), followed by 11 U (n = 11; 22%), and 10 U (n = 7; 14%). Doses of >13 U were administered in 14 patients (28%), all of whom had Grade 2 GFL at baseline. Dose selection for the glabella was left to the discretion of the treating dermatologist. Published literature advises that dosage depends on the area, muscle mass, and sex [14] and should be tailored to the individual pattern and strength of muscle activity [5,6]; since recommended doses differ between products and populations [15], as unit potency is formulation-specific, such figures are not directly transferable to Stunnox™.

## Results

Participant flow

Of 179 individuals screened, 102 were excluded (age >40 years, n = 46; wrinkle grade >2, n = 34; unable to attend follow-up, n = 12; BMI >35 kg/m², n = 10) and 77 were enrolled and treated. Twenty-seven participants (35.1%) did not complete follow-up: they were lost to follow-up at week 1 (n = 14), week 8 (n = 11), and week 12 (n = 2), because of travel or work commitments (n = 24) or relocation (n = 3) (Figure 1). The remaining 50 participants (64.9%) completed the 20-week follow-up and constitute the analysis population (completers-only). Efficacy data from the 27 noncompleters were not available for analysis, so any difference between completers and non-completers could not be assessed.

Demographics and baseline characteristics

Fifty patients completed the 20-week study (26 males, 24 females). Table 1 summarizes baseline demographics and wrinkle severity. Mean age was 32.7 ± 5.7 years (range: 21-40), with males slightly older (mean 34.2 ± 4.5 years) than females (31.0 ± 6.4 years).

At baseline, 18 patients (36%) had GFL Grade 1 and 32 (64%) had GFL Grade 2. For FL, 41 patients (82%) had Grade 1 and 9 (18%) had Grade 2. Males demonstrated higher mean baseline GFL severity (1.81 vs. 1.46 for females), while females had marginally higher baseline FL severity (1.25 vs. 1.12 for males).

Primary efficacy outcome: response at week 1

At week 1, 100% of patients (n = 50; 95% CI 92.9-100%) achieved complete resolution of both GFL and FL. Mean GFL FWS declined from 1.64 ± 0.48 to 0.00 ± 0.00 (p < 0.0001). Mean FL FWS decreased from 1.18 ± 0.39 at baseline to 0.00 ± 0.00 (p < 0.0001) (Figure 2). The first post-treatment assessment was at week 1, so the exact time of onset of action cannot be determined from this data. Representative clinical images are shown in Figure 3.

**Figure 2:**
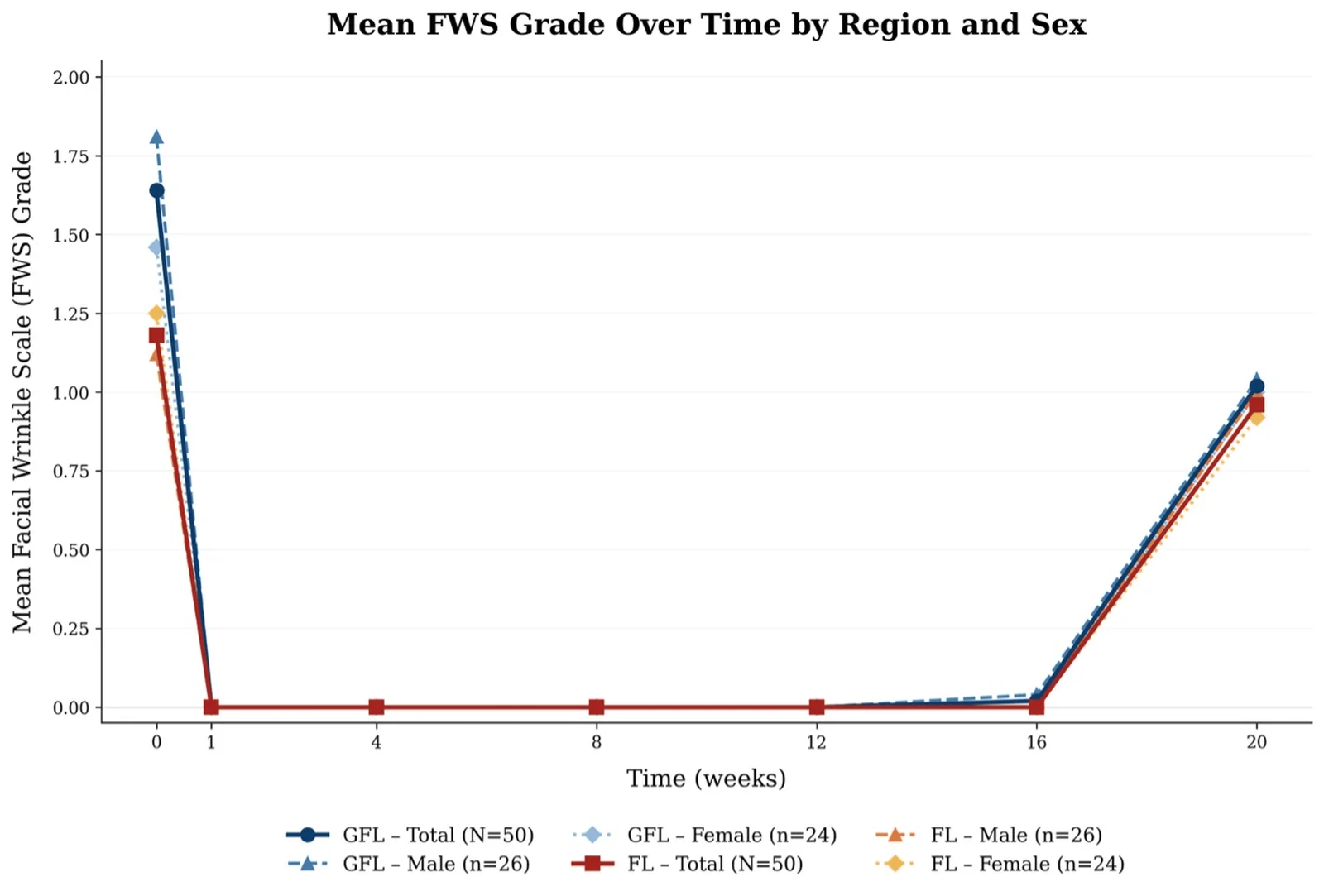
Mean FWS grade over time by anatomic region and sex FWS: Facial Wrinkle Scale; GFL: glabellar frown lines; FL: forehead lines Combined and sex-stratified mean FWS trajectories are shown for glabellar frown lines. Both regions achieved complete resolution (Grade 0) by week 1, sustained through week 12 (GFL) and week 16 (FL), with partial, nonbaseline recurrence by week 20. Sex-stratified curves paralleled the total cohort throughout, with no significant between-sex difference at week 20

**Figure 3:**
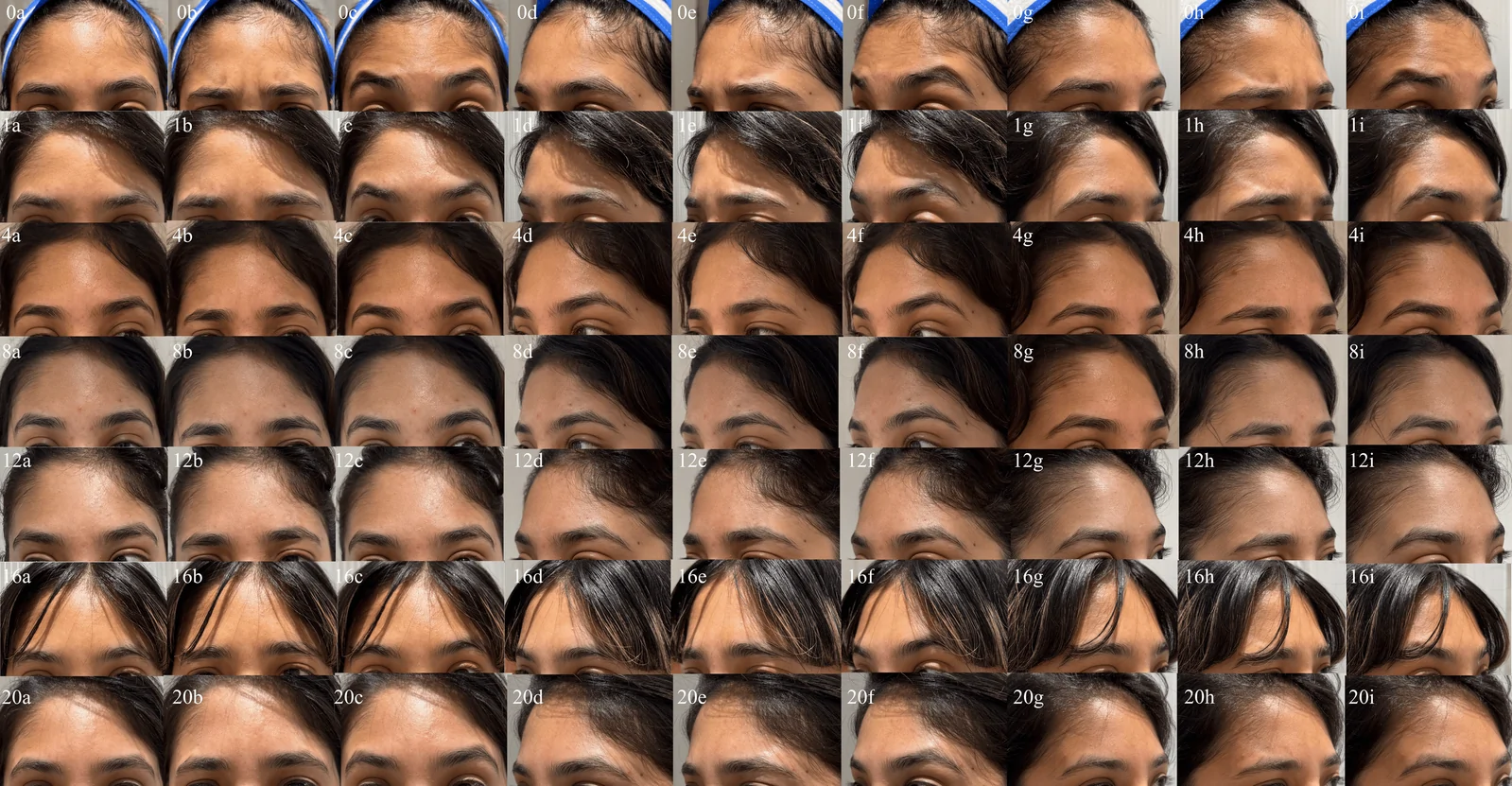
Representative image of a case at baseline and follow-up at 0, 1, 4, 8, 12, 16, and 20 weeks

Duration of effect

Complete resolution was maintained from week 1 through week 12 for GFL, and through week 16 for FL. At week 16, 49 of 50 patients (98%) remained at GFL Grade 0; one male patient (37 years, Grade 2 at baseline) recurred early to Grade 1. All patients remained at FL Grade 0 at week 16.

By week 20, partial recurrence was observed in most patients. For GFL, 49 patients (98%) had returned to Grade 1, and 1 patient (2%) to Grade 2; mean GFL FWS at week 20 was 1.02 ± 0.14 (95% CI: 0.98-1.06), significantly below baseline (p < 0.0001). For FL, 48 patients (96%) had returned to Grade 1 and 2 patients (4%) remained at Grade 0 at week 20, with mean FL FWS at week 20 of 0.96 ± 0.20 (95% CI: 0.90-1.02), significantly below baseline (p < 0.001). None exceeded their baseline severity grade at any time point (Table 2, Figure 4).

**Table 2:** FWS grade distribution at each time point for glabellar and forehead regions (N = 50) GFL: glabellar frown lines; FL: forehead lines; FWS: Facial Wrinkle Scale; SD: standard deviation Test statistic and p-value in the last two columns reflect a Wilcoxon signed-rank test comparing FWS grade at each time point against baseline (week 0), performed separately for GFL and FL as labelled. The test statistic (W, the sum of negative-rank differences) is 0.00 at every post-baseline time point because every patient's FWS grade was at or below their own baseline value (i.e., no patient scored worse than baseline at any assessment), leaving no negative-rank contribution to the statistic

Time point	GFL Grade 0 n (%)	GFL Grade 1 n (%)	GFL Grade 2 n (%)	GFL mean ±SD	FL Grade 0 n (%)	FL Grade 1 n (%)	FL Grade 2 n (%)	FL mean ± SD	Test	p-value
									statistic	
Baseline	0 (0%)	18 (36%)	32 (64%)	1.64 ± 0.48	0 (0%)	41 (82%)	9 (18%)	1.18 ± 0.39	Reference	Reference
Week 1	50 (100%)	0 (0%)	0 (0%)	0	50 (100%)	0 (0%)	0 (0%)	0	GFL: 0.00	GFL: <0.0001
									FL: 0.00	FL: <0.0001
Week 4	50 (100%)	0 (0%)	0 (0%)	0	50 (100%)	0 (0%)	0 (0%)	0	GFL: 0.00	GFL: <0.0001
									FL: 0.00	FL: <0.0001
Week 8	50 (100%)	0 (0%)	0 (0%)	0	50 (100%)	0 (0%)	0 (0%)	0	GFL: 0.00	GFL: <0.0001
									FL: 0.00	FL: <0.0001
Week 12	50 (100%)	0 (0%)	0 (0%)	0	50 (100%)	0 (0%)	0 (0%)	0	GFL: 0.00	GFL: <0.0001
									FL: 0.00	FL: <0.0001
Week 16	49 (98%)	1 (2%)	0 (0%)	0.02 ± 0.14	50 (100%)	0 (0%)	0 (0%)	0	GFL: 0.00	GFL: <0.0001
									FL: 0.00	FL: <0.0001
Week 20	0 (0%)	49 (98%)	1 (2%)	1.02 ± 0.14	2 (4%)	48 (96%)	0 (0%)	0.96 ± 0.20	GFL: 0.00	GFL: <0.0001
									FL: 0.00	FL: 0.001

**Figure 4:**
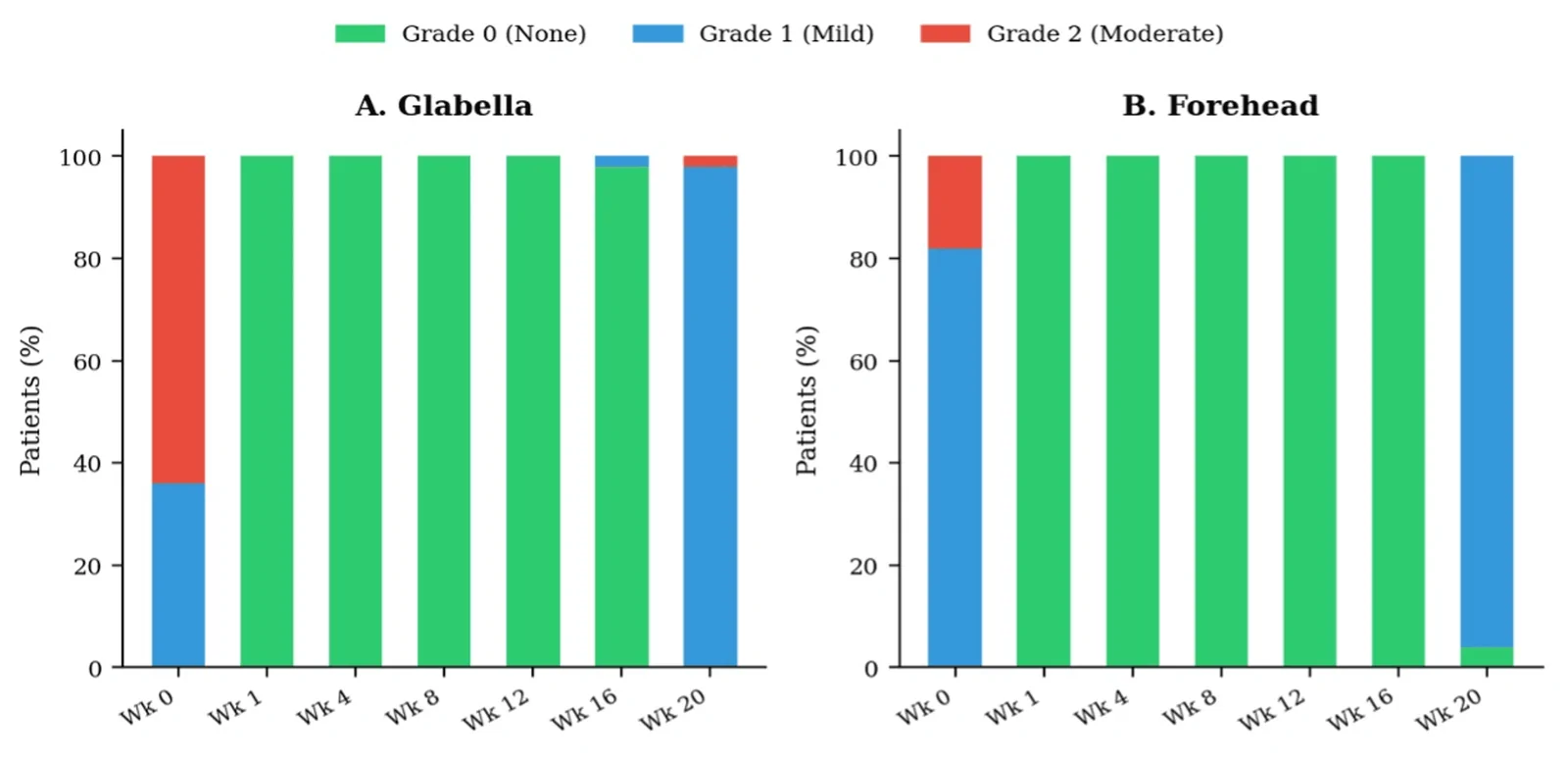
Stacked bar graphs showing the percentage distribution of FWS grades (Grade 0 = none; Grade 1 = mild; Grade 2 = moderate) at each time point for (A) glabellar frown lines and (B) forehead lines

Using the responder definition of at least a one-grade improvement from baseline [12,13], 50 of 50 patients (100%; 95% CI: 92.9-100%) were responders for both GFL and FL at weeks 1 to 16, whereas at week 20 the responder rate was 62% (31/50; 95% CI: 47.2-75.3%) for GFL and 22% (11/50; 95% CI: 11.5-36.0%) for FL. Accordingly, by week 20, 19 patients (38%) for GFL and 39 patients (78%) for FL had returned to their baseline grade. 

Gender-stratified analysis

Males had significantly higher baseline GFL severity (mean 1.81 Vs 1.46), consistent with their higher representation among Grade 2 cases (81% vs. 46%). Both genders achieved 100% Grade 0 resolution by week 1 through week 12; a single male showed recurrence at week 16. At week 20, mean GFL FWS scores were comparable between males (1.04) and females (1.00). For FL, females had slightly higher baseline severity (1.25 vs. 1.12), and marginally better at week 20 (mean: 0.92 vs. males 1.00), though this difference was not statistically significant (p = 0.14).

Safety

No serious adverse events occurred over 20 weeks. Mild injection-site erythema and transient edema were noted in three patients following injection, resolving within 24-48 hours. No ptosis, brow asymmetry, diplopia, or systemic effects were documented. Four female and one male patient (all grade I FL) experienced "heaviness" lasting four weeks without clinical ptosis. None of the patients who dropped out of the study experienced side effects at follow-up contact. 

## Discussion

This prospective study evaluated Stunnox™, a BoNT-A formulation (Gufic Biosciences Ltd., India), for Grade 1-2 GFL and FL in Indian adults with Fitzpatrick skin types III-V. Complete wrinkle resolution was observed in 100% of patients by week 1 for both regions, a finding consistent with the known pharmacodynamic profile of BoNT-A, which typically produces clinical effects within 3-7 days [2,16]. As the first post-treatment assessment was at week 1, the study cannot define the exact time of onset of action.

The duration of complete resolution, 12 weeks for GFL and week 16 for FL, can only be placed in context indirectly: published BoNT-A studies of glabellar lines generally follow responses for 3-4 months after a single treatment [12,13,17], and in a prospective open-label study in Chinese adults, responder rates (at least one-grade FWS improvement at maximum frown) were 72-82% at day 120 [13]. In the present study, all 50 completers were responders through week 16, falling to 62% (GFL) and 22% (FL) at week 20. These comparisons are indirect: the studies differ in population, product, dose, rating scale, and response definition, and Stunnox™ has not been compared head-to-head with any other BoNT-A product, so they provide context only and do not establish similar potency or interchangeable dosing. Within the present study, the longer durability observed for FL relative to the glabella may reflect the fixed 20 U Stunnox™ dose to the forehead, a comparatively higher per-muscle-unit Stunnox™ dose to the occipitofrontalis than the individualized glabellar protocol, a within-product comparison unaffected by cross-product considerations.

A clinically important finding is the pattern of week 20 recurrence. Although the majority of patients returned to Grade 1 at week 20, the mean FWS values (GFL 1.02; FL 0.96) remained significantly below their respective baseline values (1.64 and 1.18), and no patient exceeded their pretreatment wrinkle severity at any time point, a finding relevant to retreatment planning and patient counseling. However, by week 20, 19 of 50 patients (38%) for GFL and 39 of 50 (78%) for FL had returned to their baseline grade, and a one-grade improvement was retained in 62% and 22%, respectively; the significant difference in mean FWS at week 20 therefore reflects a small residual effect, mainly in patients with Grade 2 wrinkles at baseline.

Our individualized Stunnox™ glabellar dosing strategy, 9 to 17 U based on baseline severity, is consistent in principle with published guidance that favors tailored, patient-specific dosing according to muscle mass, sex, and contraction pattern [4-6,14]. Recommended doses differ between products and populations (e.g., 8 U across three glabellar points in an Asian consensus [15]), and because unit potency is formulation-specific, no unit-for-unit comparison with other products is intended or implied. A plausible rationale is that grade 1 wrinkles reflect lower-amplitude muscular contraction that may be adequately neutralized with lower doses, reducing risk of hypercorrection, brow ptosis, or an expressionless appearance. In this uncontrolled study, complete resolution at week 1 was observed in all Grade 1 and Grade 2 patients after severity-based dosing (mean 10.7 U and 13.3 U, respectively). However, because there was no fixed-dose comparator and dose selection was left to the injector, the study cannot show that individualization was superior to fixed dosing or that the doses used were optimal; these exploratory findings should inform future dose-finding studies, particularly in toxin-naïve and younger patients. For the forehead, a published dose-ranging study found that higher doses of BoNT-A (16, 32, and 48 U) produced greater efficacy and a longer duration of effect [11]; the fixed 20 U used here lies toward the lower end of that range, although unit potency differs between products. 

Gender-stratified analysis revealed that males had significantly higher baseline GFL severity (1.81 vs. 1.46), consistent with published data suggesting greater corrugator muscle mass in men [1]. Despite this, both sexes showed comparable responses, with no statistically significant between-sex difference at week 20. However, the study was not designed or powered to demonstrate equivalence, so a sex-independent effect cannot be concluded. Four female and one male participant, with Grade 1 FL at baseline, reported self -limited brow heaviness lasting for four weeks. Given the predominance among Grade 1 FL patients and female participants, a dose- and muscle mass-related mechanism is plausible, wherein the fixed 20 U forehead dose produced a proportionally greater frontalis relaxation effect in patients with milder baseline wrinkling and lower frontalis muscle bulk, manifesting as a subjective heaviness without objective ptosis.

South Asian Fitzpatrick III-V skin types remain considerably underrepresented in published BoNT-A trials [4,15] despite a large and growing consumer base in aesthetic dermatology. They may also exhibit distinct structural characteristics, including higher dermal melanin content and different collagen architecture that may influence healing and wrinkle dynamics, although these factors are unlikely to affect the pharmacological response to BoNT-A. Indian patients, like other Asian populations, are reported to have smaller facial muscles than European populations, and there is no consensus on the optimal dose per injection site [4]. 

With reference to preventive (preemptive) use, our cohort was young (mean age: 32.7 years; the 18 patients with Grade 1 GFL had a mean age of 27.6 ± 4.5 years), and Grade 1 GFL resolved at week 1 with doses of 9-13 U. However, this study did not assess whether early treatment prevents the development of static rhytids: patients were followed after a single treatment cycle, there was no control group, and no static-wrinkle outcome was measured. Evidence that early treatment delays static lines is currently limited and heterogeneous [7], and our observations should not be interpreted as evidence of prevention. Whether repeated early treatment could reduce baseline severity over multiple cycles is a hypothesis for future longitudinal studies.

Short-term tolerability of Stunnox™ in this study was favorable. No serious adverse events occurred, and mild, transient injection-site reactions resolved within 48 hours without intervention. The absence of ptosis, a feared complication of glabellar BoNT-A injection [18], consistent with the technique and dose tailoring employed, is in line with the well-established safety profile of BoNT-A in cosmetic applications [19]. The adverse events observed, namely, mild, transient local reactions and brow heaviness, are consistent in nature with the mild-to-moderate adverse events described in a published meta-analysis of studies of upper facial lines [20]. 

Limitations

This study is limited by being a single-arm, open-label, single-center study with no comparator, so causal inference is limited, and Stunnox™ cannot be compared with other products. Only 50 of 77 participants (64.9%) completed follow-up, and only completers were analyzed; thus, efficacy and duration of effect may be overestimated. Grading was done by a single trained dermatologist from photographs, blinded to dose and follow-up duration. The week 1 first assessment and four-weekly visits prevent exact determination of onset and recurrence. Glabellar doses were individualized without a fixed-dose comparator, so the optimal dose cannot be inferred. The study was not powered for sex or other subgroup comparisons. Follow-up covered one treatment cycle in adults aged 18-40 years with Fitzpatrick III-V skin and Grade 1-2 wrinkles. The findings need larger, controlled, blinded studies with longer follow-up and repeated cycles of treatment.

## Conclusions

In this single-arm, open-label, single-center study of 50 Indian adults (Fitzpatrick phototypes III-V) who completed follow-up, a single treatment with Stunnox™ was associated with complete resolution of Grade 1-2 GFL and FL at the first assessment (week 1), maintained to week 12 (glabella) and week 16 (forehead), with partial nonbaseline recurrence at week 20. An individualized glabellar dosing strategy based on baseline severity showed promising early efficacy and favorable short-term tolerability, avoiding overtreatment; a similar approach for the FL may help avoid the minimal side effects noted in five patients. The absence of serious adverse events and favorable short-term tolerability support Stunnox™ as an effective BoNT-A option for upper-face dynamic rhytids in Indian dermatological practice, providing a foundation for prospective comparative trials and dose-optimization research in South Asian populations.

## Appendices

Additional information

Funding: This study was investigator-initiated and funded by Gufic Biosciences Limited (Mumbai, India), the manufacturer of Stunnox™. The sponsor had no role in the study design, patient recruitment or screening, treatment protocol, data collection, statistical analysis, interpretation of results, or the decision to prepare, review, or submit this manuscript; these activities were performed independently by the investigators.

Conflicts of interest: The third author is an employee of Gufic Biosciences Limited, the manufacturer of the study product (Stunnox™). 

## References

[1] Kim SB, Kim HM, Ahn H, Choi YJ, Hu KS, Oh W, Kim HJ (2021). Anatomical injection guidelines for glabellar frown lines based on ultrasonographic evaluation. Toxins (Basel).

[2] Zhang X, Cai L, Yang M, Li F, Han X (2020). Botulinum toxin to treat horizontal forehead lines: a refined injection pattern accommodating the lower frontalis. Aesthet Surg J.

[3] Carruthers A, Carruthers J, De Boulle K, Lowe N, Lee E, Brin MF (2023). Treatment of crow's feet lines and forehead lines with Botox (onabotulinumtoxinA): development, insights, and impact. Medicine (Baltimore).

[4] Kamat A, Quadros T (2019). An observational study on glabellar wrinkle patterns in Indians. Indian J Dermatol Venereol Leprol.

[5] Sundaram H, Signorini M, Liew S (2016). Global aesthetics consensus: botulinum toxin type A-evidence-based review, emerging concepts, and consensus recommendations for aesthetic use, including updates on complications. Plast Reconstr Surg.

[6] Braccini F, Catoni I, Belfkira F (2023). SAMCEP Society consensus on the treatment of upper facial lines with botulinum neurotoxin type A: a tailored approach. J Cosmet Dermatol.

[7] Marinelli G, Inchingolo AD, Trilli I (2025). Proactive aesthetic strategies: evaluating the preventive role of botulinum toxin in facial aging. Muscles.

[8] Binder WJ (2006). Long-term effects of botulinum toxin type A (Botox) on facial lines: a comparison in identical twins. Arch Facial Plast Surg.

[9] Shenoy C, Agrawal R, Chandrashekar BS, Lalchandani R (2023). Comparison of safety and efficacy of two brands of botulinum toxin A for the treatment of lateral canthal lines (crow’s feet): a split-face study. J Cutan Aesthet Surg.

[10] Carruthers A, Carruthers J, Said S (2005). Dose-ranging study of botulinum toxin type A in the treatment of glabellar rhytids in females. Dermatol Surg.

[11] Carruthers A, Carruthers J, Cohen J (2003). A prospective, double-blind, randomized, parallel-group, dose-ranging study of botulinum toxin type a in female subjects with horizontal forehead rhytides. Dermatol Surg.

[12] Sattler G, Callander MJ, Grablowitz D (2010). Noninferiority of incobotulinumtoxinA, free from complexing proteins, compared with another botulinum toxin type A in the treatment of glabellar frown lines. Dermatol Surg.

[13] Wu Y, Lu Z, Xie Y (2018). OnabotulinumtoxinA treatment of moderate to severe glabellar lines in Chinese subjects after laser therapy: a prospective, open-label, noncomparative study. J Cosmet Laser Ther.

[14] Shetty MK (2008). Guidelines on the use of botulinum toxin type A. Indian J Dermatol Venereol Leprol.

[15] Ahn BK, Kim YS, Kim HJ, Rho NK, Kim HS (2013). Consensus recommendations on the aesthetic usage of botulinum toxin type A in Asians. Dermatol Surg.

[16] Carruthers JD, Carruthers JA (1992). Treatment of glabellar frown lines with C. botulinum-A exotoxin. J Dermatol Surg Oncol.

[17] Kane MA, Gold MH, Coleman WP 3rd (2015). A randomized, double-blind trial to investigate the equivalence of incobotulinumtoxinA and onabotulinumtoxinA for glabellar frown lines. Dermatol Surg.

[18] Sethi N, Singh S, DeBoulle K, Rahman E (2021). A review of complications due to the use of botulinum toxin A for cosmetic indications. Aesthetic Plast Surg.

[19] Vartanian AJ, Dayan SH (2005). Complications of botulinum toxin A use in facial rejuvenation. Facial Plast Surg Clin North Am.

[20] Brin MF, De Boulle K, Liew S (2024). Safety and tolerability of onabotulinumtoxinA in the treatment of upper facial lines from global registration studies in 5298 participants: a meta-analysis. JAAD Int.

